# The Pho regulon: a huge regulatory network in bacteria

**DOI:** 10.3389/fmicb.2015.00402

**Published:** 2015-04-30

**Authors:** Fernando Santos-Beneit

**Affiliations:** Centre for Bacterial Cell Biology, Institute for Cell and Molecular Biosciences, Medical School, Newcastle University, Newcastle upon TyneUK

**Keywords:** phosphate, Pho regulon, PHO box, regulatory networks, pathogenesis, secondary metabolism

## Abstract

One of the most important achievements of bacteria is its capability to adapt to the changing conditions of the environment. The competition for nutrients with other microorganisms, especially in the soil, where nutritional conditions are more variable, has led bacteria to evolve a plethora of mechanisms to rapidly fine-tune the requirements of the cell. One of the essential nutrients that are normally found in low concentrations in nature is inorganic phosphate (Pi). Bacteria, as well as other organisms, have developed several systems to cope for the scarcity of this nutrient. To date, the unique mechanism responding to Pi starvation known in detail is the Pho regulon, which is normally controlled by a two component system and constitutes one of the most sensible and efficient regulatory mechanisms in bacteria. Many new members of the Pho regulon have emerged in the last years in several bacteria; however, there are still many unknown questions regarding the activation and function of the whole system. This review describes the most important findings of the last three decades in relation to Pi regulation in bacteria, including: the PHO box, the Pi signaling pathway and the Pi starvation response. The role of the Pho regulon in nutritional regulation cross-talk, secondary metabolite production, and pathogenesis is discussed in detail.

## Introduction

Phosphorus, in terms of cellular content, is the fifth most important element. It is essential for several important biological processes such as the inheritance of genetic materials, energy metabolism, membrane integrity, and intracellular signaling. The availability of this nutrient has many medical, agricultural, and pharmaceutical implications. Misregulation of phosphorus can cause serious human disorders like tumor-induced osteomalacia, muscle myopathy, cardiomyopathy, neuropathy, and haemolysis, and in some cases, it can contribute to death (Bergwitz and Jüppner, 2011). In agriculture, phosphorus is required to ensure high levels of agricultural productivity (Ha and Tran, 2014). With this aim, modern agricultural practice extensively uses fertilizers, in many cases producing negative environmental effects. From an industrial point of view, phosphorous availability is also quite important. Pharmaceutical companies commercialize an impressive number of antibiotics every year and other valuable secondary metabolites such as antifungal compounds, anti-tumor agents, immuno-suppressants, and pigments (Hopwood, 2007). The production of most of these compounds is quite reduced by high amounts of phosphorous in the fermentation tanks (Martin, 2004). Therefore, companies are often forced to use low concentrations of this nutrient which results in a significant decrease of the growth yields.

The main assimilable form of phosphorous in bacteria is the orthophosphate anion (PO_4_^3-^), most commonly known as inorganic phosphate (Pi). Despite the essentiality of Pi for life, this nutrient is usually found at very low concentrations in the environment, especially in soil. To adapt and survive to Pi scarcity, bacteria, as well as yeasts, plants and animals, have evolved physiological and biochemical responses aimed to acquire and save this nutrient. Whether these mechanisms in eukaryotes involve similar or different bacterial systems is unknown. This review provides a critical overview of the most important insights regarding Pi regulation in bacteria, for example antibiotic production, nutritional stress networking, or pathogenesis.

## The Pho Regulon and the PHO Box

The Phosphate (Pho) regulon is a global regulatory mechanism involved in bacterial Pi management that was first characterized in *Escherichia coli*, and later in many other bacterial species (Wanner and Chang, 1987). The most common members activated by the Pho regulon are: extracellular enzymes capable of obtaining Pi from organic phosphates, Pi-specific transporters, and enzymes involved in storage and saving of the nutrient. The Pst Pi-specific transporter is the most conserved member of the Pho regulon in all bacteria. Other Pi transporters commonly found in bacteria, such as the low affinity Pi transporter Pit, are regulated in a variable fashion manner in the different species (Santos-Beneit et al., 2008). *E. coli* activates additional transporters for phosphorous-containing compounds, such as Ugp (i.e., glycerophosphodiesters uptake) and Phn (i.e., phosphonates uptake). Among the Pi scavengers, the alkaline phosphatases (PhoA), phospholipases (PhoD), glycerophosphodiester phosphodiesterases (GlpQ and UgpQ), phytases (PhyC) and 5′-nucleotidases (UshA) are the most common enzymes induced in response to Pi starvation in bacteria (Wanner and Chang, 1987). For the storage of Pi, most bacteria induce the expression of PpK, which is able to accumulate polyphosphate as a Pi reservoir and, when needed, reuse it (Ghorbel et al., 2006a). For saving nutrients, some bacteria are able to replaced teichoic acids (Pi-rich polymers found in the cell wall of Gram-positive bacteria) by teichuronic acids (Pi-free polymers). The biosynthetic genes are repressed and activated, respectively, by the Pho regulon (Liu et al., 1998).

The Pho regulon is controlled by a two-component regulatory system which comprises an inner-membrane histidine kinase sensor protein and a cytoplasmic transcriptional response regulator. These proteins have received different names in some bacteria, such as: PhoR–PhoB in *E. coli* (Tommassen et al., 1982), PhoR–PhoP in *Bacillus subtilis* (Hulett et al., 1994), PnpR–PnpS in *Streptococcus pneumoniae* (Novak et al., 1999), PhoR–PhoS in *Corynebacterium glutamicum* (Kocan et al., 2006), PhosS–PhosR in *Campylobacter jejuni* (Wösten et al., 2006), SphS–SphR in *Synechocystis* sp. (Juntarajumnong et al., 2007), and SenX3–RegX3 in *Mycobacterium smegmatis* (Glover et al., 2007). In all cases, upon Pi scarcity, the response regulator is phosphorylated on an aspartic residue by the sensor kinase. The phosphorylated response regulator is able to bind to specific sequences on the DNA and activate or repress the transcription of genes. These specific sequences, known as PHO boxes, were first characterized in *E. coli* (Makino et al., 1988). Initially, the PHO boxes were defined as 18-nucleotide sequences comprising two 7-nucleotide direct repeat units separated by four non-conserved nucleotides. However, after the crystallographic studies by Blanco et al. (2002), the PHO box was defined as the sum of two 11-nucleotide direct repeat units composed of seven well conserved and four less conserved nucleotides in each. Similar features have also been observed in many other bacteria, among others: *B. subtilis*, *Sinorhizobium meliloti, C. glutamicum,* and *Streptomyces coelicolor* (Hulett, 1996; Yuan et al., 2006; Schaaf and Bott, 2007; Sola-Landa et al., 2008). The consensus sequence of the PHO box varies from one species to another and depends on the specific contacts that the amino acids of the response regulator are able to eject with the bases of the DNA (Blanco et al., 2002). For instance, the most conserved motif of the PHO box in *E. coli* is CTGTCAT (Yoshida et al., 2012) while in *S. coelicolor* is GTTCACC (Sola-Landa et al., 2008); this is not strange since the two microorganisms have a distinct GC content in its genomes. Nonetheless, the PHO boxes of diverse promoters in a specific bacterium contain distinct DNA sequences, which results in different protein-DNA binding affinities. This has been well documented in *S. coelicolor,* in which a high degree of variation in the sequence conservation, number, and organization of the different direct repeat units that form the PHO boxes has been noticed (Martín et al., 2012). According to this variation, the Pho operators in this bacterium have been classified as simple or complex operators. It is important to note that several experimentally confirmed PHO boxes are found in genes that lack an obvious Pi regulation (at least under a given condition) and that, on the contrary, clear Pho regulon members lack any conserved PHO boxes (Allenby et al., 2012; Martín et al., 2012). This might indicate the existence of additional features in the PHO boxes and/or extra regulatory proteins or ligands involved in the control of the PHO regulon. In this sense, the interaction of the response regulator with the RNA polymerase sigma subunit has been well reported for triggering transcription initiation in both *E. coli* and *B. subtilis*. In *E. coli,* the σ^70^ subunit is involved in this interaction, while at least two different sigma factors (σ^A^ and σ^E^) may be involved in *B. subtilis* (Makino et al., 1993; Paul et al., 2004). Recently, Blanco et al. (2011) have provided crystallographic evidence that PhoB directly contacts the C-terminal region of σ^70^ to accomplish RNA polymerase recruitment in *E. coli*.

**FIGURE 1:**
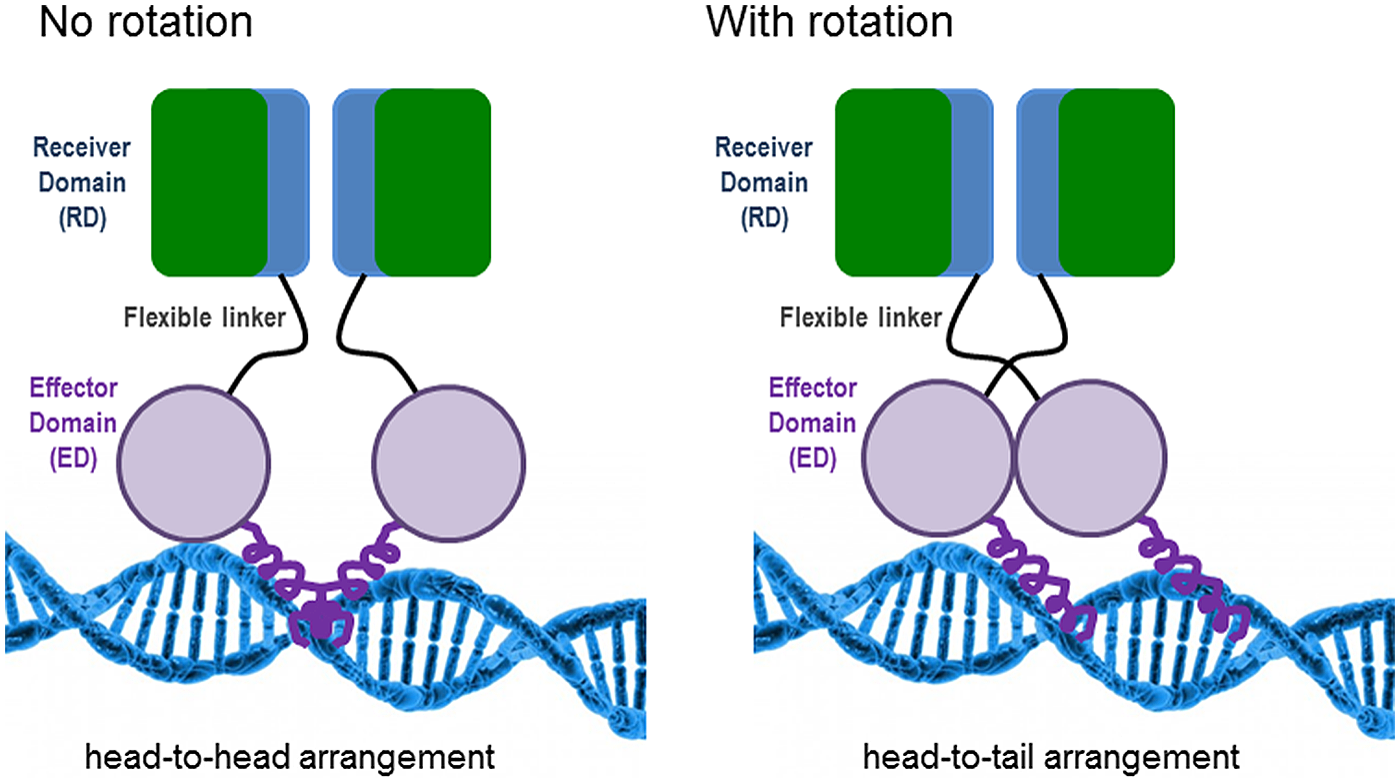
**Model of PhoB binding to the DNA in *Escherichia coli* according to the work of Bachhawat et al. (2005).** The non-rotational arrangement would provide an inactive state by positioning the effector domains (EDs) in opposite directions, incompatible with tandem DNA binding. In the rotational arrangement, the receiver domains (RDs) would form a twofold symmetric dimer, while the EDs would bind to DNA with tandem symmetry.

The features for dimerization and oligomerization of PhoB (*E. coli*) and PhoP (*B. subtilis*) on the DNA differ. In *E. coli*, PhoB is already a dimer before binding to the DNA, but it has to be phosphorylated in order to become active (Bachhawat et al., 2005). The oligomerization of the active dimers on the DNA is achieved in a hierarchical and cooperative manner and is favored by protein–protein interactions (Blanco et al., 2012). PhoB presents two structural domains connected by a flexible linker: (i) the N-terminal receiver domain (RD) containing the conserved aspartate (Asp53) to which a phosphoryl group is transferred from the histidine (His215) of PhoR and (ii) the C-terminal effector domain (ED), which presents a winged-helix fold with the specific DNA binding and transactivation properties (Solá et al., 1999). Phosphorylation of the RD is needed to switch on the ED, although if the RD is removed, the ED can constitutively bind to the DNA (Ellison and McCleary, 2000). Different structural analyses have determined that the RD dimerises with twofold rotational symmetry (i.e., head-to-head). This orientation contrasts with the binding of the ED to the direct repeats units of the PHO boxes in a tandem head-to-tail orientation (Blanco et al., 2002). Bachhawat et al. (2005) have proposed a model which implies an obligatory rotation of the flexible linker connecting the RD and ED domains when they are in their active state; avoiding the problem of rotation (see **Figure 1**). In *B. subtilis*, PhoP dimerises asymmetrically by using two distinct faces of the RD, being able to form a great variety of multimers on the DNA (Chen et al., 2003).

## Pi Sensing and Signal Transduction Pathway

Inorganic phosphate depletion in the medium has been shown to be essential for activation of the Pho regulon in bacteria; differing with other microorganisms such as *Saccharomyces cerevisiae,* in which the intracellular Pi concentration seems to account for this activation (Auesukaree et al., 2004). The complete signal transduction system that senses and transmits the Pi scarcity signal to the response regulator has not been yet completely elucidated and seems to vary from one organism to another.

In *E. coli*, the Pi-sensing pathway requires, apart from the two-component system PhoR–PhoB, five additional proteins. Four of those are components of the Pi-specific transporter Pst (PstSCAB) and the other is the metal binding protein, PhoU. Under Pi limitation, PhoB is activated by PhoR acting as a kinase, but under Pi-replete conditions, PhoB activation is interrupted by PhoR acting as a phosphatase (Carmany et al., 2003). PhoU is required for PhoB dephosphorylation under Pi-rich conditions in an as yet unknown manner. When *phoU* is mutated or deleted, PhoR behaves as a constitutive PhoB kinase, leading to high expression of the Pho regulon genes (Steed and Wanner, 1993; Rice et al., 2009). PhoU is involved not only in control of the autokinase activity of PhoR, but also in the control of the Pst system in order to avoid an uncontrolled Pi uptake that could be toxic for the cell (Surin et al., 1986; Muda et al., 1992; Carmany et al., 2003; Gardner et al., 2014). Whether it is PstSCAB, PhoR, PhoU, or another player the component that senses the Pi scarcity is still unknown. Since PhoR does not contain a significant periplasmic sensory domain, it makes sense to assume that the Per-Arnt-Sim (PAS) domain of the protein senses a cytoplasmic signal; although the nature of the signal is not known.

Curiously, although *phoU* is found in many bacterial genomes, *B. subtilis* lacks this gene. The Pi signal transduction network in this bacterium includes a positive feedback loop between the PhoP–PhoR and ResD–ResE two-component systems (Schau et al., 2004). Alternative players such as the Spo0B–Spo0A two-component system, the AbrB and ScoC transition state regulators and an intermediate of the teichoic acid synthesis pathway are also involved in this network (Hulett, 1996; Sun et al., 1996; Kaushal et al., 2010; Botella et al., 2014). ResD activates genes involved in both aerobic and anaerobic respiration, including two terminal oxidase genes. ResD is required for 80% of the wild-type *B. subtilis* Pho response. This transcriptional regulator does not bind to the *phoPR* operon and seems to eject its control through the expression of terminal oxidases (such as *ctaA* or cytochrome *bd*) that are able to regulate the autophosphorylation activity of PhoR, although the mechanism of this control is not known. PhoP induces the expression of *resDE*, indicating a feedback loop between these two component systems (Birkey et al., 1998).

Disruption of the *abrB* transition state regulator causes a 20% reduction of the wild-type *B. subtilis* Pho response. Actually, a *resD*–*abrB* double mutant shows no Pho induction and resembles the phenotype of a *phoPR* mutant strain (Hulett et al., 1994). The other transition state regulator involved in the network (ScoC), contrary to AbrB, produces a negative effect on the expression of *phoPR* (Kaushal et al., 2010). This negative effect is accumulative with the one ejected by another *B. subtilis* regulator, CcpA, which is involved in carbon metabolism (Puri-Taneja et al., 2006; see below). Spo0B–Spo0A attenuates the Pho response by negative regulation of both ResE–ResD and AbrB pathways (Strauch et al., 1990; Prágai et al., 2004), connecting the control of *B. subtilis* sporulation (i.e., Spo0B–Spo0A) with the Pi response. Recently, Botella et al. (2014) have shown new insights in the activation of the *B. subtilis* Pho system. These authors have reported that the PhoR autokinase activity is inhibited by an intracellular intermediate of teichoic acid biosynthesis which is sensed through the cytoplasmic PAS domain of the protein. These two mechanisms of Pi signal transduction (i.e., *E. coli* and *B. subtilis*) may not be fully conserved in other bacteria. For example, no constitutive induction of the Pho regulon has been displayed in *pst* and *phoU* mutants of several *Streptomyces* species (Díaz et al., 2005; Ghorbel et al., 2006b). On the other hand, as mentioned above, several cross-talks among two-component systems have been reported in *E. coli* and *B. subtilis*. Actually, at least seven different kinases are able to phosphorylate PhoB in *E. coli* (Amemura et al., 1990; Fisher et al., 1995; Zhou et al., 2005). This promiscuity of the Pho system is not observed in some bacteria, for example *S. coelicolor*, where PhoR appears to be the only specific sensor kinase able to phosphorylate PhoP (Fernández-Martínez et al., 2012).

## Involvement of Pi Regulation in Secondary Metabolite Production

The synthesis of many classes of secondary metabolites (i.e., macrolides, tetracyclines, aminoglycosides, etc.) is negatively regulated by high Pi concentrations in the culture media (reviewed in Martin, 2004). Pi control has been profoundly studied in species of the *Streptomyces* genus, which provides the source of most of the antibiotic compounds in clinical use today (Hopwood, 2007). Most of these studies were performed a long time ago and report data of the overall Pi concentration effect on the control of antibiotic production, but surprisingly the molecular mechanism of this control remains still unclear. Works showing absent or retarded antibiotic production when high Pi concentrations are added to the culture medium have been performed at least in the following *Streptomycetes*: *S. griseus* (Martin and Demain, 1976), *S. clavuligerus* (Aharonowitz and Demain, 1977), *S. fradiae* (Vu-Trong et al., 1981), *S. noursei* (Müller et al., 1984); *S. rosa* (Masuma et al., 1986), *S. coelicolor* (Doull and Vining, 1990), *S. tendae* (Hege-Treskatis et al., 1992), *S. acrimycini* (Asturias et al., 1994), *S. hygroscopicus* (Cheng et al., 1995), *S. rimosus* (McDowall et al., 1999), *S. lividans* (Chouayekh and Virolle, 2002), *S. venezuelae* (Jakeman et al., 2006), *S. natalensis* (Mendes et al., 2007), *S. cacaoi* (Tenconi et al., 2012), and *S. tsukubaensis* (Martínez-Castro et al., 2013). Contrary to the large amount of studies describing the effect of the Pi concentration on antibiotic production, very few exist about the implication of the Pho regulon in this process. The effect of *phoP* deletion in the control of antibiotic production has been determined so far in only three bacteria: *S. lividans* (Sola-Landa et al., 2003), *S. natalensis* (Mendes et al., 2007), and *S. coelicolor* (Santos-Beneit et al., 2009a; Fernández-Martínez et al., 2012; Thomas et al., 2012). Sola-Landa et al. (2003) reported for the first time the involvement of the PhoR–PhoP system in the control of secondary metabolite production. These authors showed that synthesis of the antibiotics actinorhodin and undecylprodigiosin is greatly enhanced in both Δ*phoP* and Δ*phoRP S. lividans* mutants in R5 media (either solid or liquid). A similar result is obtained in R5 medium for the same antibiotics in *S. coelicolor* Δ*phoP*, a close relative of *S. lividans* (Fernández-Martínez et al., 2012), or in the pimaricin producer, *S. natalensis*, using another complex medium, NBG (Mendes et al., 2007). However, the effect of PhoR–PhoP on antibiotic production is culture media-dependent. For example, actinorhodin and undecylprodigiosin productions are repressed in a *S. coelicolor* Δ*phoP* mutant when using, instead of complex media, defined starch-containing media (Santos-Beneit et al., 2009a; Fernández-Martínez et al., 2012; Thomas et al., 2012).

**FIGURE 2:**
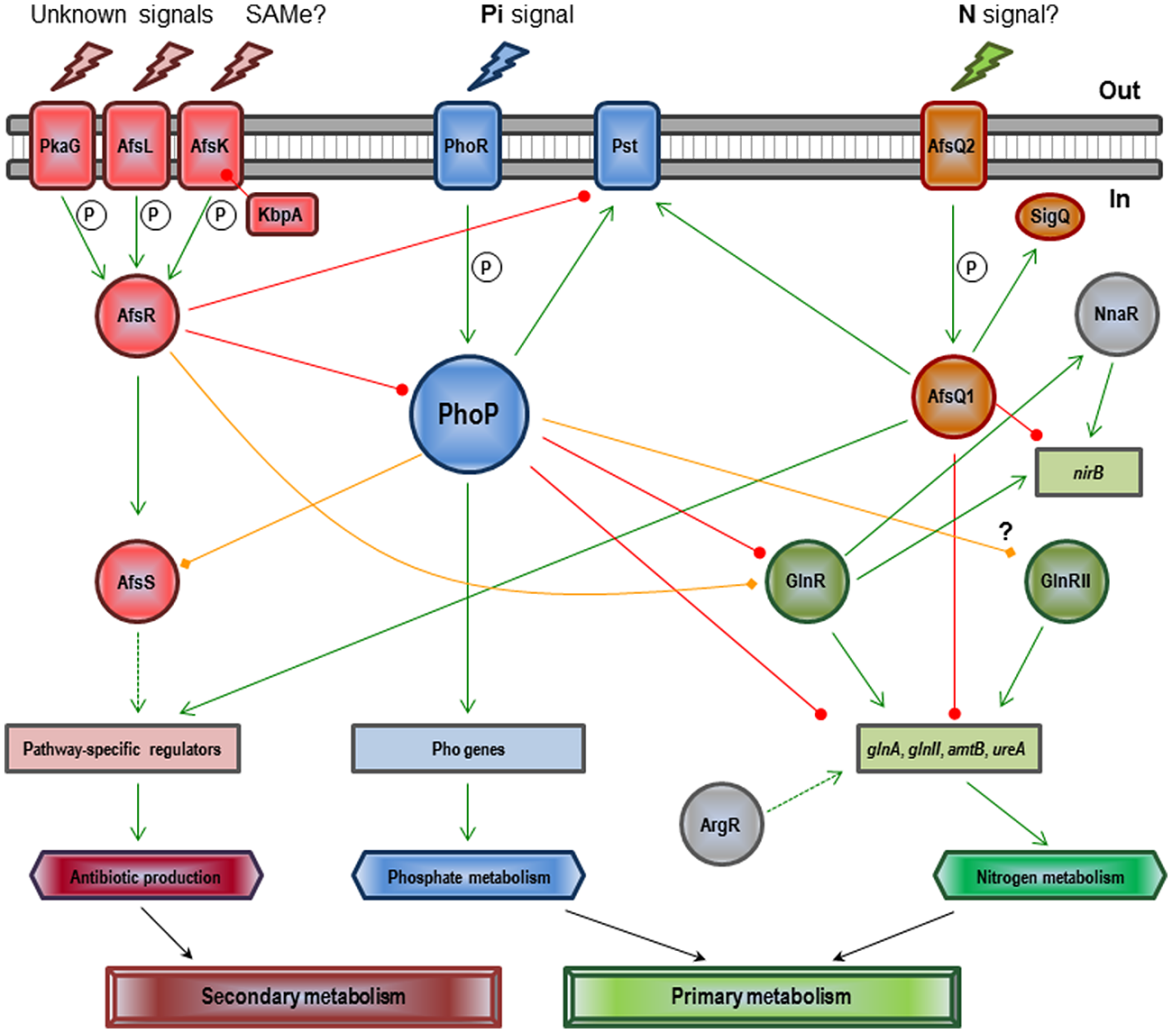
**Regulatory network involving PhoP and other *Streptomyces coelicolor* global regulators in the control of Pi and N metabolisms and antibiotic production (see text).** Green arrows indicate induction. Circle-ended red lines indicate repression. Rhombus-ended orange lines indicate dual activation/repression or unknown regulation. Dashed lines indicate indirect or uncharacterized links. P indicates activation by phosphorylation. SAMe (S-adenosyl-L-methionine), Pi (inorganic phosphate), and N (nitrogen).

The regulatory mechanism of PhoR–PhoP on antibiotic control is unknown. Only four antibiotic regulatory genes (*afsS*, *atrA*, *cdaR,* and *scbR*) have been described as members of the Pho regulon in a chromatin-immunoprecipitation-on-microarray analysis of *S. coelicolor* wild-type and Δ*phoP* mutant cultures using purified PhoP protein (Allenby et al., 2012). Unfortunately, detailed studies on this control only exist in the *S. coelicolor afsS* gene (Santos-Beneit et al., 2009a, 2011). AfsS is part of a signal transduction system which includes additional components, such as AfsR, AfsK, AfsL, PkaG, and KbpA (see **Figure 2**). Upon sensing particular signals, such as the accumulation of S-adenosyl-L-methionine (SAM), AfsR is phosphorylated by the serine/threonine kinase AfsK (Matsumoto et al., 1994; Jin et al., 2011). The KbpA protein is able to inhibit AfsK autophosphorylation (Umeyama et al., 2002). Two additional serine/threonine kinases (AfsL and PkaG) can also phosphorylate AfsR (Sawai et al., 2004), which suggests that AfsR integrates multiple signals. The phosphorylated active form of AfsR binds the -35 region of the *afsS* promoter and activates its transcription (Lee et al., 2002; Tanaka et al., 2007). The fate of the small protein AfsS is not known, but appears to interact with the RNA-polymerase activating the transcription of the pathway specific antibiotic regulatory genes. PhoP competes for binding to the *afsS* promoter region with AfsR (Santos-Beneit et al., 2009a). Both regulators behave as competitive activators on *afsS*, with each regulator being able to activate the transcription of the gene when acting separately, but acting as competitive proteins when occurring together (Santos-Beneit et al., 2011). Each regulator has its own binding requirements and recognizes specific DNA sequences. AfsR is able to bind to the promoters of other Pho regulon members, such as *pstS* and *phoRP* (i.e., establishing a nice cross-talk between these two proteins). This kind of interaction may allow streptomycetes to fine-tune secondary metabolism in relation to different signals, like for example Pi scarcity and SAM accumulation. In general, it can be conclude that the effect of the Pi concentration on antibiotic production is much more conserved and severe than the Pho-dependent effect, which means that unknown additional systems may account for the Pi antibiotic production control.

## Nutritional Stress Networking

Bacterial systems involved in carbon (C), nitrogen (N), and Pi metabolisms must interact for coordination, but the mechanisms involved in these interactions are just starting to be understood. This section focuses mainly on the interactions of Pi regulation with those of C and N, although other nutritional interconnections are also described (see **Table 1**).

**Table 1:** Studies showing different inorganic phosphate (Pi) cross-talks in bacteria.

Nutrients	Regulators	Regulated genes	Bacterium	Reference
Pi, C	PhoP, CcpA	*phoPR, phoA*, *phoB*	*Bacillus subtilis*	Choi and Saier (2005), Puri-Taneja et al. (2006)
Pi, C	PhoB, CRP	*ugpBAECQ, psiE*	*Escherichia coli*	Kasahara et al. (1991), Kim et al. (2000)
Pi, C	PhoB, SgrR	*sgrS*	*E. coli*	Richards and Vanderpool (2012)
Pi, C	PhoS, GlxR	*pstS*	*Corynebacterium glutamicum*	Panhorst et al. (2011)
Pi, C	PhoP, unknown	*pstS, glpQ1, glpQ2*	*Streptomyces coelicolor*	Díaz et al. (2005), Santos-Beneit et al. (2009b)
Pi, C, Fe	PhoB, CRP, RosR, RirA	*pssA*	*Rhizobium leguminosarum*	Janczarek and Urbanik-Sypniewska (2013)
Pi, N	PhoP, TnrA	*ykoL*	*B. subtilis*	Robichon et al. (2000)
Pi, N	*PhoP*, *GlnR*, *GlnRII*, *AfsR*, *AfsQ1*, *NnaR*, *ArgR*	*glnR*, *glnA*, *glnII*, *amtB*, *urea*, *nirB*, *pstS*	*S. coelicolor*	Rodríguez-García et al. (2009), Santos-Beneit et al. (2012), Wang et al. (2013), full references in text.
Pi, S	SphR?	*sulfate transporters*	*Microcystis aeruginosa*	Harke and Gobler (2013)
Pi, As	PhoB, AioR, ArsR	*aioBA, phoB, pstS*	*Agrobacterium tumefaciens*	Kang et al. (2012)
Pi, Fe	PhoB, Fur	*phoB*, *pstS*, *phoU*, *esrC*	*Edwardsiella tarda*	Chakraborty et al. (2011)
Pi, Na	PhoP, NhaC	*phoPR*	*B. subtilis*	Prágai et al. (2001)
Pi, K, N	EIIA^Ntr^, PhoRB, KdpDE	*kdpFABC*, *pstSCAB*	*E. coli*	Lüttmann et al. (2012)

### Pi and C Cross-Talk

Several works have reported the regulation of Pho regulon members by different C sources as well as by different known C regulators, but only some describe this regulation at the molecular level. One of the most interesting studies describes the direct involvement of CCR (carboepression) in the expression of the *phoPR* operon in *B. subtilis* (Puri-Taneja et al., 2006). This operon is repressed by direct binding of CcpA (a transcriptional regulator mediating CCR) to a *cre* box located immediately upstream of the PHO box. In the same direction, Choi and Saier (2005) showed that CcpA influences expression of other Pho regulon genes in *B. subtilis* such as the alkaline phosphatase genes, *phoA,* and *phoB*. These authors showed that the effect of glucose on these two genes depends not only on CcpA but also on the growth conditions, including Pi and N sources. Similar interactions have been also shown in other bacteria, for example *Vibrio vulnificus* (Oh et al., 2007). Another example of Pi and C interaction is the regulation of the *ugp* operon in *E. coli*, which is induced under both Pi- and C-limiting conditions from two separate promoters. The downstream promoter has multiple PHO boxes while the upstream promoter has a consensus sequence for the cAMP–CRP complex, which is formed under the presence of non-preferred carbon sources (Kasahara et al., 1991). A similar control involving these two regulatory pathways has also been described for *psiE* in *E. coli* (Kim et al., 2000). In *C. glutamicum*, the global cAMP-dependent transcriptional regulator, known as GlxR, has been shown to bind to the *pstS* promoter and activate its transcription under Pi-limiting conditions in a C source-dependent manner (Panhorst et al., 2011). The existence of additional Pho-dependent promoters controlled by the C source has been reported in three *S. coelicolor* genes: *pstS*, *glpQ1,* and *glpQ2* (Díaz et al., 2005; Esteban et al., 2008; Santos-Beneit et al., 2009b).

Cross-regulation in the other direction (i.e., Pi to C) has been also documented. For example, a connection between the glucose-Pi stress response (mediated by the SgrR and SgrS regulators) and the Pi metabolism has been reported in *E. coli*. Direct induction of the Pho regulon is sufficient to partially suppress the glucose-Pi stress growth defect of a *sgrS* mutant; although the molecular mechanism is yet unclear (Richards and Vanderpool, 2012). Another example of Pi affecting C metabolism has been shown in *S. coelicolor* in which PhoP is able to repress glycogen catabolism and gluconeogenesis by means of the regulation of key enzymes of these pathways (Thomas et al., 2012). A more complex network involving C and Pi regulations has been described in a recent work with the soil bacterium *Rhizobium leguminosarum*. In this study, among the different nutrients tested, iron, C, and Pi have been found to strongly affect transcription of the *pssA* gene (involved in the first step of exopolysaccharide synthesis). The regulation of this gene has been shown to be very complex, involving at least three different promoters and the binding of at least four different regulators, RosR, RirA, cAMP–CRP, and PhoB; responding to flavonoids (and other environmental factors), iron, C, and Pi, respectively, (Janczarek and Urbanik-Sypniewska, 2013). Overall, these results reflect a coordinated redevelopment of metabolism in many different bacteria to balance the availability of Pi with C utilization.

### Pi and N Cross-Talk

It has long been known that N sources and Pi content regulate microbial growth through interconnected networks (Doull and Vining, 1990). One of the first interactions described at the molecular level was the link between the glutamate and Pi positive effects over the *ykoL* gene in *B. subtilis*. The gene is induced by both PhoP and TnrA regulators (Robichon et al., 2000). TnrA is involved, together with GlnR, CodY, and SigL, in the control of N metabolism in response to N supply (Fisher, 1999). A link between Pi and N regulations has been also described in *E. coli*. In this bacterium, the transcription of *phoB,* and many other Pho regulon members is up-regulated under N limitation (Marzan and Shimizu, 2011). On the other hand, putative PHO boxes have been detected in several genes involved in *E. coli* N metabolism, such as those of the glutamine permease operon *glnHPQ* and the *glnLG* two-component system that regulates the glutamine synthetase *glnA* gene (Merrick and Edwards, 1995; Rodríguez-García et al., 2009).

The interplay between Pi and N regulatory pathways reaches its maximum complexity in *S. coelicolor* (see **Figure 2**). Under Pi limitation, PhoP represses transcription of the major N regulator, *glnR*, as well as that of *glnA*, *glnII*, *amtB,* and *ureA* (all GlnR targets) by direct binding to their promoters; actually, PhoP and GlnR compete for binding at least in *glnA*, *glnII,* and *amtB* promoters (Tiffert et al., 2008; Rodríguez-García et al., 2009; Wang et al., 2012; Sola-Landa et al., 2013). Interestingly, the PhoP binding site in *glnA* overlaps not only with GlnR but also with another regulator, AfsQ1; existing three global regulators competing for the same promoter (Wang et al., 2013). AfsQ1 is the response regulator of a two component system (AfsQ2–AfsQ1) which controls antibiotic production and N, C, and Pi uptake (Shu et al., 2009). The network is even more complex: besides GlnR, AfsQ1, and PhoP, the regulators GlnRII, NnaR, AfsR, and ArgR are also involved. GlnRII is a second N regulator which binds to most of the GlnR targets, although there is no agreement as to whether it is controlled by PhoP or not (Rodríguez-García et al., 2009; Allenby et al., 2012). NnaR, in cooperation with GlnR, regulates several N assimilation genes including *nirB*, which is also controlled by AfsQ1 (Amin et al., 2012; Wang et al., 2013). The secondary metabolite regulator AfsR binds to the promoter region of *glnR* and overlaps with the binding site of PhoP (Rodríguez-García et al., 2009; Santos-Beneit et al., 2012). Finally, the arginine regulator ArgR is needed for optimal *glnA* expression (Pérez-Redondo et al., 2012). In general, under conditions of Pi limitation, PhoP represses genes required for N assimilation because it is unnecessary without a supply of cellular Pi.

### Pi Cross-Talk with other Nutrient Stresses

Inorganic phosphate cross-talk with other nutrients has been also reported. For example, under low Pi conditions, some cyanobacteria substitute phosphorus with sulfur within their membrane lipids (Van Mooy et al., 2006). In fact, Pi limitation has been shown to increase the transcription of sulfate transporters genes in the cyanobacterium *Microcystis aeruginosa* (Harke and Gobler, 2013). Another example is the tight co-regulation existing between arsenic and Pi metabolisms in *Agrobacterium tumefaciens*. This interaction involves the Pi suppression of arsenite oxidation and the arsenite enhancing of Pi stress response (Kang et al., 2012). The physiological explanation for this cross-talk is complex and might involve other mechanisms that allow the microbial cell to react to Pi-limiting situations and avoid the toxic biochemical effects of arsenic. In *S. meliloti,* several genes coding for iron transporters have been described as members of the Pho regulon (Yuan et al., 2006). Alternatively, iron has been proved to be an important modulator of the activity of Pho-regulated phosphatases in *Pseudomonas fluorescens*, although this control is not at the transcriptional level (Monds et al., 2006). More interesting is the cross-talk between Pho and Fur systems in the bacterial pathogen *Edwardsiella tarda* (Chakraborty et al., 2011). In this bacterium, the ferric uptake regulator Fur senses changes in iron concentration and regulates the expression of type III and VI secretion systems through the EsrC regulator. Under low Pi concentrations, PhoB suppresses the secretion, and expression of proteins from the type III and VI secretion systems, either directly or indirectly through *esrC* control. On the other hand, under high iron concentration, Fur suppresses the transcription of *phoB*, *pstSCAB*–*phoU,* and *esrC* in an indirect manner via unknown regulators, perhaps involving an interaction between Fur and PhoU proteins (Chakraborty et al., 2011). This cross-talk is of biological significance because iron regulation is crucial for controlling the pathogenicity of many bacteria. Another example of links between nutrients is the influence of sodium in the expression of the *phoPR* operon of *B. subtilis* by means of the NhaC regulator. In this bacterium, several genes of the Pho regulon have been shown to be overexpressed in the absence of NhaC (dependent on the external sodium concentration) and repressed when the protein is overproduced, in accordance with the negative effect on growth of NhaC (Prágai et al., 2001). Finally, an interesting link involving at least three different nutrients (potassium, N, and Pi) has been described in *E. coli* (Lüttmann et al., 2012). In this bacterium, the co-sensing protein EIIA^Ntr^, a member of the so-called nitrogen-related phosphotransferase system (PTS^Ntr^), has been shown to modulate the activities of two distinct sensor kinases, KdpD (involved in regulation of potassium uptake) and PhoR. EIIA^Ntr^ interacts with both proteins and stimulates their kinase activity, as shown by *in vivo* and *in vitro* experiments (Lüttmann et al., 2012). The two component system KdpD–KdpE positively controls the synthesis of the high-affinity potassium transporter KdpFABC under low concentration of this nutrient (Lüttmann et al., 2009); and PhoR–PhoP activates the synthesis of the high-affinity Pi transporter PstSCAB under low Pi concentration. Therefore, EIIA^Ntr^ globally stimulates both high-affinity potassium and Pi transports; although regulations of Kdp and Pho regulons are independent (i.e., potassium and Pi regulations do not cross-talk).

### Pi Starvation and General Stresses

Interaction of the Pho regulon with other stresses such as the oxidative, osmotic, acid, and cell wall stresses has been also documented (see next section). For example, several studies in distinct bacteria have reported the Pho-dependent up-regulation of genes coding for catalases that protect bacteria from the oxidative stress by degrading hydrogen peroxide (Yuan et al., 2005). Alternatively, different works have brought to light links between the Pho regulon and the synthesis of polyphosphate and guanosine tetraphosphate (ppGpp). Polyphosphate is a known stress response molecule which is very important for the stationary-phase survival of bacteria and is synthesized in a Pho-dependent manner when environmental stresses occur (Kornberg et al., 1999; Shiba et al., 2000). On the other hand, ppGpp is a key phosphate containing molecule involved in the stringent response, which is responsible for the inhibition of RNA synthesis when there is a deficiency in the amino acids availability (Chatterji and Ojha, 2001). The Pho regulon is repressed in *E. coli* mutants that cannot accumulate ppGpp, such as *relA* and *spoT* mutants, whereas ppGpp content is altered in *pst* mutants (Loewen et al., 1998; Spira and Yagil, 1998). In addition, some *pstA–pstB* mutants have been shown to alter the accumulation of RpoS; a sigma factor implicated in the cellular response to many environmental stresses (Schurdell et al., 2007). RpoS is highly unstable in exponentially growing cells, whereas its stability increases dramatically upon ppGpp accumulation (Gentry et al., 1993) and under certain stress conditions such as Pi starvation (Bougdour et al., 2006) or magnesium starvation (Bougdour et al., 2008). The small protein IraP accounts for RpoS stabilization during Pi starvation conditions by blocking the action of RssB, an adaptor protein for RpoS degradation (Bougdour et al., 2006). Interestingly, the *iraP* promoter is positively regulated by ppGpp (Bougdour and Gottesman, 2007). All these results demonstrate that the Pi cross-talk with nutrient and general stresses is indicative that all stresses regulation might have common features associated to stress responses, for example associated to RpoS regulon, as well as features specific to the applied stress.

## The Pho Regulon is Involved in Pathogenesis

Bacterial pathogens, including those of humans, animals, and plants, deal with Pi-limiting or Pi-rich environments in the host depending on the site of infection. Although the main function of the Pho regulon is the control of the Pi homeostasis, this system also plays a role in pathogenesis. However, the mechanisms connecting the Pho regulon with pathogenesis are not always well defined. This section focuses in a small part of the numerous reports that showed a relationship between Pi regulation and pathogenesis. Most of them were performed in *V. cholerae*, *Pseudomonas* spp. and pathogenic *E. coli* species (see reviews of Lamarche et al., 2008; Crépin et al., 2011; Chekabab et al., 2014). There are several strategies accounting for virulence in which the Pho regulon has been shown to be involved. The main ones are: (a) tolerance to acidity, (b) toxin production, (c) biofilm formation, and (d) resistance to antimicrobial compounds.

(a) In some bacteria, the Pho system is able to respond to external acidity by controlling the transcription of genes important for acid shock resistance. One example is the PhoB-dependent activation of the acid-inducible *asr* gene in *E. coli*. The Asr protein has a role in protecting periplasmic proteins from the deleterious effects of low pH environments, such as those of the stomach or intestine, indicating a direct role of PhoB in resistance to acidity (Suziedeliene et al., 1999; Gajiwala and Burley, 2000).
(b) One of the clearest examples of the involvement of the Pho regulon on toxin production is reported in *V. cholera*. In this bacterium, the regulatory cascade leading to the production of TCP and CT toxins is repressed by PhoB under Pi-limiting conditions. However, under the normal high Pi conditions of the gut, the regulatory cascade is not repressed, being able to activate TCP and CT productions (Pratt et al., 2010). Another example has been described in *Pseudomonas aeruginosa*, although in the opposite direction. In this pathogen, the production of the pyocyanin toxin is positively activated by PhoB. This is not strange, because infection with Gram-negative pathogens normally leads to Pi reduction in the plasma to a suboptimal level for bacterial growth. In this manner, it has been shown that a host supplemented with excess Pi is less susceptible to *P. aeruginosa* infection than the non-supplemented (Jensen et al., 2006; Zaborin et al., 2012). This result suggests that the opportunistic pathogen *P. aeruginosa* can use Pi deficiency as an environmental signal of host trauma to shift from non-virulent to lethal phenotypes. In a similar way, it has been shown that activation of the Pho regulon induces a shift of *Bacteroides fragilis* from gut symbiosis to pathogenicity (Wakimoto et al., 2013).
(c) Biofilms are mixed microbial populations typically embedded in a matrix of extracellular polymers that lead to a number of functions, including: concentration of virulence factors, tolerance to environmental stresses, resistance to antimicrobial agents, redox regulation, nutrition, adhesiveness, locomotion, etc. (Flemming and Wingender, 2010). A link between Pi homeostasis and biofilm formation has been reported in different bacteria. For example, in the plant pathogen *A. tumefaciens* biofilm formation is enhanced in low Pi conditions by PhoB (Danhorn et al., 2004). Influence of the Pho regulon on biofilm formation has been also described in *Pseudomonas*, *Proteus mirabilis*, *E. coli,* and *V. cholerae* (Monds et al., 2001, 2007; O’May et al., 2009). In *P. aeruginosa*, Pi limitation promotes bacterial hyper-swarming through PhoB induction of rhamnolipid biosurfactants (Blus-Kadosh et al., 2013). On the other hand, the published data on enteric pathogens suggest a regulatory model in which biofilm formation would be promoted during the course of infection, whereas under Pi-limiting conditions (such as those of the aquatic environments outside of the host), PhoB would shut down biofilm formation, and promote bacterial survival and dissemination (Pratt et al., 2009; Yoshida et al., 2010).
(d) The lower susceptibility of biofilm-grown bacteria to antimicrobial agents, such as antibiotics and biocides, has been widely investigated (Mah, 2012). However, involvement of Pi regulation in antibiotic resistance by distinct mechanisms to those related with biofilms has been also reported. For example, Pi concentration has been shown to regulate to a high extent vancomycin resistance in two different streptomycetes: *S. coelicolor* and *S. lividans* (Santos-Beneit and Martín, 2013). Interestingly, an *S. coelicolor* mutant in a PhoP down-regulated gene (putatively involved in exopolysaccharide synthesis) has been shown to be at least ten times more resistant to vancomycin than the wild type strain (Santos-Beneit et al., 2014). In *Enterococcus faecium*, PhoB can be activated by the sensor kinase VanS (the partner of the VanR response regulator, which is involved in the induction of vancomycin resistance genes), pointing to a cross-talk between the Pi starvation response and the vancomycin resistance pathways in this bacterium (Fisher et al., 1995). In other work, it has been shown that *pst* mutants of pathogenic *E. coli* strains display an increased sensitivity to vancomycin and different cationic antimicrobial peptides (Lamarche et al., 2008). Something similar has been described in *M. tuberculosis* when the *phoU* homolog gene *phoY2* is inactivated (Shi and Zhang, 2010).

Therefore, understanding Pi regulation in pathogens and/or in hosts may be of great value for the development of new drugs.

## Concluding Remarks (Perspectives)

The physiological response to Pi-starvation in bacteria is a good example for systems biology studies, not only because it constitutes a fast and very sensible responding system, but also because it generates an amazing regulatory network with few precedents in bacteria, involving primary and secondary metabolisms, virulent factors, and multiple cross-talks in some cases. Different transcriptomic analyses and other works have brought to light the following general observations for the Pi regulatory network in bacteria:

(a) In most bacteria, the Pho response controls many more genes than just those related to Pi metabolism; revealing a surprisingly diverse role for the system, not only as a regulator of Pi assimilation, but also as a “master” regulator of the cell.
(b) Many different levels of Pho-dependent regulation are possible, including up-regulation, down-regulation, and complex regulatory patterns. Actually, the number of genes negatively regulated (directly or indirectly) is normally larger than the number of activated genes (Martín et al., 2012). Two different requirements of the response regulator may account for this difference: (i) the response regulator needs to be phosphorylated in order to binding tightly to the DNA and/or form strong dimers and multimers (Allen et al., 2001; Bachhawat et al., 2005). (ii) The response regulator must be able to interact with RNA polymerase in order to activate transcription (Makino et al., 1993; Chen et al., 2004). These requirements are not essential for transcriptional repression, for which a very weak contact can hamper the biding or movement of RNA polymerase or the binding of a transcriptional activator (Browning and Busby, 2004). This difference explains why many genes can be slightly regulated by the Pho system before Pi depletion; although transcriptional activation only occurs under Pi starvation (i.e., when the response regulator is in its phosphorylated state). This fact is clearly observed in *V. cholerae*, in which the Pho system is required for full growth and normal stress response, not only under Pi limiting conditions but also under Pi abundance (Lery et al., 2013).
(c) The total number of genes involved in the Pi-limitation response is expected to be considerably larger than those regulated just by PhoP, pointing to alternative systems of Pi regulation in bacteria. For example, two Pho-independent systems (one being the σ^B^ general stress regulon and the other remaining unknown) have been shown to be involved in the *B. subtilis* Pi starvation response (Antelmann et al., 2000; Prágai and Harwood, 2002). In addition, up to three two-component systems (PhoR1–PhoP1, PhoR2–PhoP2, and PhoR3–PhoP3) and an orphan response regulator (PhoP4) have been shown to be responsible for the Pi regulation response in the bacterium *Myxococcus xanthus* (Moraleda-Muñoz et al., 2003; Pham et al., 2006). Finally, two hierarchic response systems to Pi scarcity have been proposed in the marine cyanobacterium *Synechococcus* sp. WH8102, the first being the typical PhoR–PhoB system and the second depending on the CRP family transcriptional regulator PtrA (Ostrowski et al., 2010).

Advances in the field of Pi regulation promise to be very important for new developments in biotechnology. Therefore, future work should focus on the complete characterization of the mechanism systems involved in bacterial Pi regulation (either novel or depending on the Pho system); investing greater effort on those related to secondary metabolite production control and pathogenesis. These systems may include other players distinct to proteins (or additional), such as small non-coding RNAs, metabolites, and precursors.

## Conflict of Interest Statement

The author declares that the research was conducted in the absence of any commercial or financial relationships that could be construed as a potential conflict of interest.
